# Exploring Surgical Strategies for Uterine Fibroid Treatment: A Comprehensive Review of Literature on Open and Minimally Invasive Approaches

**DOI:** 10.3390/medicina60010064

**Published:** 2023-12-28

**Authors:** Stefano Cianci, Ferdinando Antonio Gulino, Vittorio Palmara, Marco La Verde, Carlo Ronsini, Paola Romeo, Sara Occhipinti, Giosuè Giordano Incognito, Vito Andrea Capozzi, Stefano Restaino, Giuseppe Vizzielli, Marco Palumbo

**Affiliations:** 1 Unit of Gynecology and Obstetrics, Department of Human Pathology of Adult and Childhood “G. Barresi”, University of Messina, 98122 Messina, Italy; docferdi@hotmail.it (F.A.G.); vittorio.palmara@unime.it (V.P.); paolaromeo135@gmail.com (P.R.); 2 Department of Woman, Child and General and Specialized Surgery, University of Campania “Luigi Vanvitelli”, 80138 Naples, Italy; marco.laverde88@gmail.com (M.L.V.); carlo.ronsini90@gmail.com (C.R.); 3 Department of General Surgery and Medical Surgical Specialties, University of Catania, 95124 Catania, Italy; saraocchipinti91@gmail.com (S.O.); giordanoincognito@gmail.com (G.G.I.); mpalumbo@unict.it (M.P.); 4 Department of Obstetrics and Gynecology, University of Parma, 43125 Parma, Italy; capozzivitoandrea@gmail.com; 5 Clinic of Obstetrics and Gynecology, “Santa Maria della Misericordia” University Hospital, Azienda Sanitaria Universitaria Friuli Centrale, 33100 Udine, Italy; restaino.stefano@gmail.com (S.R.); giuseppevizzielli@yahoo.it (G.V.); 6 Department of Medicine, University of Udine, 33100 Udine, Italy

**Keywords:** fibroids, myomectomy, laparoscopy, endoscopy, mini-laparotomy, minimally invasive surgery, benign uterine pathology, fertility

## Abstract

*Background and Objectives:* Uterine myomas represent one of the most prevalent pathologies affecting the female population. These benign neoplasms originate from the smooth muscular cells of the uterus, and they can be either single or multiple. Often associated with debilitating symptoms such as pelvic heaviness, pain, constipation, and urinary dysfunctions, the surgical management of myomectomy exhibits considerable variability. This diversity in approaches is influenced by factors such as the number and size of myomas, the patient’s age, and overall clinical conditions. This study aims to elucidate and compare the advantages and disadvantages of different surgical approaches, specifically endoscopic procedures versus open surgery, providing valuable insights for clinical decision making. *Materials and Methods:* A comprehensive bibliographic search spanning from 2013 to 2023 was systematically conducted across databases including Medline, Embase, the Cochrane Database of Systematic Reviews, and ClinicalTrials.gov. The search utilized keywords such as “myomectomy laparoscopic and open”, “myomectomy open and minimally invasive”, “myomectomy open and laparoscopic”, and “myomectomy open vs. laparoscopic.” The research methodology, along with predetermined inclusion and exclusion criteria, was established prior to the search, ensuring a systematic and rigorous approach. Subsequently, data analysis was carried out. *Results:* Following the study selection process, 25 articles met the eligibility criteria for inclusion in this analysis. The average numbers of myomas were 3.7 (ranging from 1 to 13.7) and 5.4 (ranging from 1 to 13.5) for the minimally invasive surgery and open surgery groups, respectively. In terms of myoma size, the total averages across studies were 7 cm (ranging from 4.8 to 14) for the minimally invasive group and 8 cm (ranging from 3.9 to 11.2) for the open surgery group. The average pregnancy and delivery rates were 29.7% (ranging from 1.8 to 100) for the minimally invasive group and 28.5% (ranging from 1.8 to 100) for the open surgery group. Regarding complications, the average rate was 14.2% (ranging from 0 to 50) for the endoscopic group and 22.3% (ranging from 0 to 60.3) for the laparotomic group. *Conclusions:* In conclusion, a critical factor influencing the choice of surgical approach is primarily the size and quantity of fibroids. The mini-laparotomic approach emerges as a viable alternative to endoscopy, demonstrating favorable surgical outcomes and aesthetic results. Interestingly, the type of surgical procedure appears to have no significant impact on the pregnancy rate.

## 1. Introduction

Uterine myomas stand out as one of the most commonly diagnosed pathologies in the female population, with a prevalence reported in the literature of 80% principally in over-50-year-old patients and 50% in the reproductive age group [1].

Interestingly, only 40% of myomas are symptomatic, with the majority being asymptomatic and incidentally discovered during routine ultrasound scans [2,3].

However, just a small portion of myomas are related to symptoms, with an estimated incidence of 40%. Consequently, most of them are casually diagnosed during routine ultrasound scans [2,3].

Myomas are benign neoplasms that originate from the smooth muscular cells of the uterus and can manifest as single or multiple growths, exhibiting diverse parenchymal characteristics based on the prevalence of muscular or fibroid components. In some instances, the number and size of these neoplasms can escalate to substantial proportions, resulting in an enlarged uterine size comparable to that of a full-term pregnant uterus or even larger [4]. This condition is often associated with debilitating symptoms, including pelvic heaviness, pain, constipation, and urinary dysfunctions. Additionally, patients may experience heavy menstrual and inter-menstrual bleeding, leading to chronic and sometimes severe anemia with iron deficiency. In the fertile population, myomas can contribute to subfertility, primarily due to implantation failure. Even in pregnant women, these neoplasms may interfere with fetal growth and delivery, elevating the risk of intra- and post-partum hemorrhage [5,6]. The manifestation and clinical effects vary depending on the size, number, and position of the myomas in the uterine body.

Recognizing the diverse clinical scenarios, the International Federation of Gynecology and Obstetrics (FIGO) has established a classification system based on myoma location within the uterus and, in the case of extra-uterine neoplasms, within the abdomen [7]. This classification ranges from FIGO 0, where the myoma is located entirely in the endometrial cavity, to FIGO 8, where the myoma has no connection with the myometrium.

While hysterectomy is the primary indication for this pathology, considering age of incidence and related symptoms, for the fertile age population, the preferred surgical intervention is myoma removal with uterine preservation [8,9]. Beyond surgical considerations, medical treatments, such as oral or intra-uterine hormones, GnRH agonists, and aromatase inhibitors, may be contemplated based on the size, localization, number, and growth of myomas [10,11].

Other options, especially adopted in patients averse to surgery or fragile patients, are uterine embolization or radiofrequency ablation [12].

However, their efficacy is limited, offering only a partial reduction in myoma size and symptom relief. The complete eradication of the pathology remains achievable only through surgery, making the surgical approach the most commonly adopted worldwide [13].

### Surgical Approaches to Myomectomy

The surgical procedure performed to remove uterine fibroids, called myomectomy, is a procedure typically recommended for women of fertile age who experience symptoms as previously described or with a history of sub-fertility.

The variability in the surgical approach to myomectomy is influenced by diverse factors such as the number and size of myomas, the patient’s age, and clinical conditions. Additionally, variables such as the surgeon’s experience, surgical philosophy, hospital equipment, and patient preferences play crucial roles.

In light of these considerations, three primary surgical approaches can be discerned for the removal of myomas:

- Abdominal myomectomy: This is the most common type of surgical procedure. It involves making an incision in the abdominal wall to gain access to the uterus. This approach enables the surgeon to extract fibroids situated on the uterine surface or those deep within the uterine wall [14]. Abdominal myomectomy is typically chosen under specific circumstances, including scenarios where fibroids are notably large or numerous, deeply embedded within the uterine wall, when the uterus is enlarged due to fibroids, or when a patient has a history of multiple abdominal surgeries or significant scar tissue [15].
- Laparoscopic myomectomy: This is a minimally invasive procedure where small incisions are made in the abdominal wall through which a laparoscope (a thin, lighted tube equipped with a camera) is employed to guide the surgeon in the removal of fibroids [16]. This method typically results in shorter recovery times and less post-operative pain compared to an abdominal myomectomy [17]. Laparoscopic myomectomy is chosen in specific scenarios, such as small- to medium-sized fibroids or multiple fibroids of small to medium size, as well as for women who specifically request this approach [18].
- Robotic-assisted myomectomy: This approach shares similarities with laparoscopic myomectomy, but integrates robotic technology to augment the precision and dexterity of the surgeon’s movements, particularly during uterine suturing [19]. This surgical technique proves especially beneficial in complex cases. Robotic-assisted myomectomy is selected in situations where the advantages of robotic technology can elevate the surgical procedure, including intricate cases with large or numerous fibroids, challenging-to-reach locations, instances involving significant scar tissue or adhesions from prior surgeries, and for patients desiring fertility preservation [20].
- Hysteroscopic myomectomy: This procedure is used for submucosal fibroids that are located within the uterine cavity [21]. The hysteroscope, a thin, lighted tube, is inserted through the vagina and cervix to access the uterus. The fibroids are then removed or destroyed using specialized electrified instruments [22].

After myomectomy, the recovery times can be different even for the same surgical approaches. It is common for patients to experience some discomfort, and a period of rest and restricted activity is usually advised [23]. Depending on the type of myomectomy, patients may be able to return to normal activities within a few days to weeks [24].

While myomectomy effectively treats the symptoms associated with fibroids, it is worth noting that it does not guarantee that new fibroids will not develop in the future, especially in the fertile population [25]. For this reason, it is crucial for individuals who have undergone a myomectomy to discuss their future fertility plans with their healthcare provider, as the procedure may have implications for pregnancy [26].

Overall, a myomectomy can provide significant relief for those affected by uterine fibroids, allowing them to regain their quality of life and, in many cases, preserving their fertility [27]. It is imperative to consult with a healthcare professional to ascertain the appropriate indication and treatment plan based on individual circumstances [28].

Given these considerations, the optimal surgical approach to myomectomy remains uncertain and is subject to the individual decisions of surgeons. To address this uncertainty, we conducted a systematic literature review with the aim of providing an overview of the available data, categorizing the surgical approaches broadly into endoscopic versus open surgery. The primary objective of this study is to present evidence that illuminates the advantages and disadvantages of different surgical approaches employed in myomectomy procedures. The goal is to uncover the potentials and limitations of these approaches, offering concrete clinical and surgical insights to guide informed decision making.

## 2. Materials and Methods

A comprehensive bibliographic search spanning from 2013 to 2023 was systematically conducted across databases including Medline, Embase, the Cochrane Database of Systematic Reviews, and ClinicalTrials.gov. The search utilized keywords such as “myomectomy laparoscopic and open”, “myomectomy open and minimally invasive”, “myomectomy open and laparoscopic”, and “myomectomy open vs. laparoscopic”. The research methodology, along with predetermined inclusion and exclusion criteria, was established prior to the search, ensuring a systematic and rigorous approach. Subsequently, data analysis was carried out. We categorized laparoscopic myomectomy and robotic myomectomy procedures under the same category that we termed “minimally invasive” myomectomy (MI). We then divided patients into two groups: one comprised individuals who underwent laparotomic myomectomy, and the other included those who underwent minimally invasive myomectomy.

Only papers written in English were included. Commentaries, letters to editors, editorials, and conference abstracts were excluded. The systematic review was performed in accordance with the Preferred Reporting Items for Systematic Reviews and Meta-Analyses (PRISMA) guidelines [29]. Two authors (S.O. and G.I.) independently screened the titles and abstracts of each citation and included them for full-text review. A consensus on the relevance was obtained by mutual agreement. In the case of a discrepancy between the two researchers, a third author (S.C.) made the final decision.

**Figure 1:**
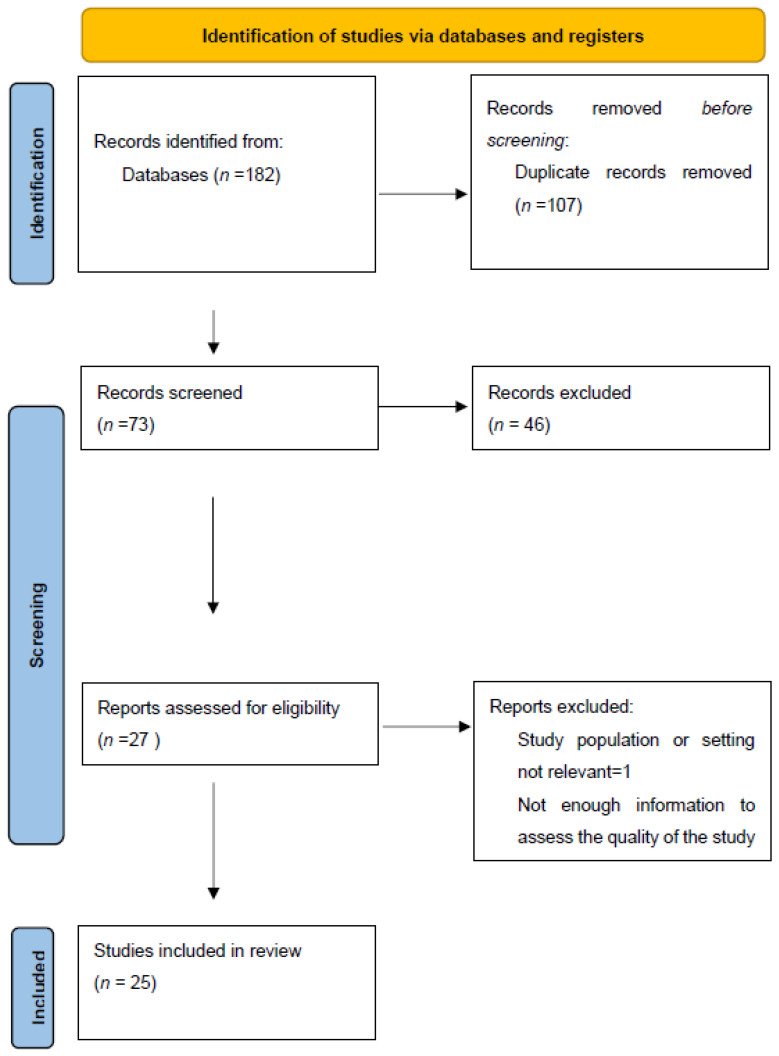
Prisma flow diagram.

Each retrieved full-text article was independently evaluated for inclusion by another author (F.A.G.). Any potential disagreement was solved by discussion with a third author (S.C.). Our systematic bibliographic research strategy identified 182 articles. After screening of abstracts and titles and the removal of 107 duplicates, 26 full-text records were assessed for eligibility. Finally, 25 studies were included in the systematic review (Figure 1).

## 3. Results

After the process of study selection, 25 articles [30,31,32,33,34,35,36,37,38,39,40,41,42,43,44,45,46,47,48,49,50,51,52,53,54] were considered eligible for the study. The strength of recommendation was level B for all the selected articles, indicating a low level of evidence. All selected papers were case–control studies aimed to compare the surgical approaches to myomectomy: open surgery vs. endoscopic surgery. A summary of the main findings of the studies is reported in Table 1.

The reported series in the different articles were variable, ranging from 3 to more than 20,000 enrolled patients. To simplify the data, we included robot-assisted and standard laparoscopy in the same category, within the minimally invasive surgery group.

The overall average size and number of myomas, as reported in studies where data were available, varied widely. Specifically, the average numbers of myomas were 3.7 (ranging from 1 to 13.7) and 5.4 (ranging from 1 to 13.5) for the minimally invasive surgery and open surgery groups, respectively. The total average sizes of the myomas reported in the studies, considered as total sizes even in cases of multiple myomas, were 7 cm (ranging from 4.8 and 14) and 8 cm (ranging from 3.9 to 11.2) for the minimally invasive surgery group and open surgery group, respectively.

Given that we included only myomectomies in the study, excluding hysterectomies, the study population was relatively young, with average ages of 37.2 for the minimally invasive group and 37.4 for the laparotomy group. The average body mass index (BMI) calculated for both groups was 23.8 in the endoscopic group and 24.6 in the open surgery group. Utilizing the available data from the studies, we calculated the average pregnancy and delivery rates, which were 29.7% (ranging from 1.8 to 100) for the minimally invasive group and 28.5% (ranging from 1.8 and 100) for the other group. Additionally, the cesarean section rates were 40% for the open surgery group and 35.5% for the minimally invasive surgery group.

Some surgical data were documented in the studies and an average across the studies was made. Data were compared between the two groups.

In particular, the overall EBL averages were 217.4 mL (ranging from 65 to 406 mL) and 378.33 mL (ranging from 100 to 1290 mL) for the minimally invasive group and open surgery group, respectively.

The complication rates presented some differences between two groups, with averages of 14.2% (ranging from 0 to 50) for the endoscopic group and 22.3% (ranging from 0 to 60.3) for the laparotomic group. The average hospital stays for the groups were 2 days (ranging from 0.58 to 6.9) for the minimally invasive group and 2.6 days (ranging from 2 to 10.3) for the open surgery group.

## 4. Discussion

The choice between a minimally invasive or laparotomic approach for a myomectomy remains a highly debated topic in gynecologic surgery. Currently, this decision is largely contingent on the surgeon’s preference, as concrete and standardized indications are lacking to guide surgeons toward the optimal surgical pathway. Both open and minimally invasive approaches present some strengths and weaknesses in the context of myomectomy procedures. Notably, factors such as the size or number of myomas are not sufficient to decide on the type of surgical approach. Some surgeons, even those skilled in endoscopic procedures, may opt for an open approach (including a mini-laparotomic incision) due to considerations such as oncological risk, procedural time, and overall surgical impact. Moreover, some argue that the aesthetic outcome could be more favorable with a small suprapubic incision compared to multiple trocar incisions in the iliac fossa. The question presents various aspects and characteristics which are open to debate as well as considerations from different perspectives.

### 4.1. Oncological Risk

One of the most critical aspects of laparoscopic myomectomy is the oncological risk, which is principally represented by uterine sarcomas. They are quite rare malignant uterine neoplasms that arise from the muscular layer and/or connective tissue of the uterus. They represent a very exiguous part of female genital malignancies, with an estimated incidence of 1% of female pelvic malignancies [55]. The core issue arises from the fact that even if they are very rare, the oncological implications can be dramatic, as this type of pathology is more aggressive than other uterine malignancies, thereby substantially affecting the survival rates of patients. The oncological risk becomes exponential for misdiagnosed patients who are subjected to endoscopic myomectomy, especially when the myoma is morcellated inside the abdomen without safety procedures such as in-bag morcellation. The dissemination of malignant cells inside the abdomen may, unfortunately, determine the patient’s death [56].

Based on this, the US Food and Drug Administration (FDA) recommends avoiding laparoscopic morcellation of myomas [57].

A solution to this issue was sought in two different ways: by preoperatively predicting the risk of malignancy for patients undergoing operative procedures and by exploring safe methods of extracting the specimens.

Several studies have presented evidence and indications aiming to minimize the potential spread of malignant cells during power morcellation, utilizing containment systems such as endo-bags and evolute endo-bags with the specific function of covering the morcellator, thus avoiding contact between the specimens being removed and the abdomen. However, there are no definitive conclusions regarding safety [58]. A recent study, for instance, assessed seven distinct containment systems featuring varied materials and characteristics to evaluate their capacity for cell spread [59]. The obtained results differed significantly between the different devices, demonstrating the necessity for a more thorough safety evaluation of in-bag extraction. Moreover, the impact of CO2 pressure on the potential risk of malignant cell spread is another aspect requiring better investigation [60].

The second crucial aspect is the accuracy of pre-operative diagnosis, which is needed in order to obtain a reliable indication of the risk of malignancy.

The first diagnostic level for uterine fibroids remains the ultrasound (US) scan. US scans, especially thanks to 3D technology, have reached a very high grade of accuracy in the evaluation of characteristics of myomas such as dimension, localization, and vascularization [61]. However, from an oncological point of view, based on the literature data, US scans have low sensitivity and specificity rates regarding differential diagnosis between uterine myomas and sarcomas. In particular, distinguishing between degenerative myomas or colliquative myomas (without malignant features) and malignant degeneration remains a challenging aspect of imaging. Consequentially, its diagnostic accuracy remains relatively low [62,63].

A more specific and accurate diagnostic method is magnetic resonance imaging (MRI). The advantages are represented by the fact that, as reported in the literature, MRI presents a higher sensitivity and specificity than US scans in sarcoma identification [64]. Moreover, MRI seems to be able to distinguish between degenerative myomas and malignant sarcoma [65]. The diagnostic foundation relies on a specific cut-off signal intensity. Novel algorithms that include serum markers such as LDH levels are available and have shown promising results in terms of differential diagnosis and sensitivity/specificity rates [66]. Even a recent meta-analysis demonstrated the high diagnostic accuracy of MRI with respect to US scans for uterine sarcoma detection [67].

While MRI might seem like the definitive solution for identifying high-risk uterine sarcoma patients and guiding surgical decisions more effectively, there are still pertinent limitations under discussion [68].

First of all, MRI is a second-level radiologic examination requiring more time than other types of radiologic technologies such as X-ray or computerized tomographic (CT) scans. Secondly, MRI examinations are more expensive than other imaging tools. Indeed, the literature presents some deficiencies in the cost-effective evaluation of MRI for pre-operatory screening of uterine sarcomas. A study by Tong et Al. [68] reported a series of 1960 patients who underwent MRI screening prior to surgery. The obtained results were very promising, as the authors recorded 100% sensitivity and 97% specificity. Even from a cost perspective, the screening program seemed to be sustainable. However, it is crucial to contextualize and evaluate these data, especially in the context of different healthcare systems. Different regions have different availabilities of MRI machines at different costs. For this reason, the MRI indication is not actually adopted for all patients. The most used clinical practice on pre-operatory work-up indications for uterine fibroid patients is the use of MRI only for suspected cases of malignant degeneration detected upon US scan. The criteria adopted to assess the malignancy risk are aberrant fibroid morphology, necrosis, hypervascularization, and rapid growth. In case of a concrete suspicion of sarcoma, the endoscopic approach should be avoided. Considering factors such as age, reproductive desire, and patient preferences, the decision to opt for a hysterectomy over a myomectomy should be carefully assessed. Counseling patients within this framework constitutes one of the pivotal steps in the therapeutic course.

### 4.2. Fertility

As reported in the literature, the presence of myomas, especially in the case of sub-mucosal myomas and or multiple large fibroids, has a negative impact on fertility rates. Indeed, it is one of the most frequent causes of sub-fertility [5]. The reasons for this, depending on the fibroid’s position within the uterus, may be associated with endometrial alterations leading to reduced implantation; changes in myometrial muscular fiber contraction; and distortion of pelvic anatomy, including tubes and ovaries [69,70]. As a result, numerous studies have confirmed that after myomectomy, the pregnancy rate increases significantly, reaching up to 50–70% [37,70,71,72,73]. However, several studies have investigated whether the type of surgical approach could have an impact on fertility rate, but the data are not in accordance among different studies. Several studies have affirmed that the pregnancy outcomes in terms of pregnancy rate and delivery rate do not differ in populations submitted to laparotomic myomectomy vs. endoscopic myomectomy. The reported reasons stem from the primary objective of eliminating the cause of subfertility, irrespective of the method employed to achieve this goal [37,71,72].

Some other evidence has suggested that the surgical approach adopted for fibroid removal could have a role in the fertility rate [73]. In this case, the hypothesis is related to the possible enhanced risk of post-operatory adherence after a laparotomic approach, which could interfere with the tubal capability to catch and transport the oocyte to the uterine cavity. However, this aspect should be contextualized, because the available literature presents series of patients that show important differences. For instance, in a substantial number of comparative series, a majority of laparotomies conducted for fibroid removal involved predominantly large myomas, often infiltrating the endometrial cavity more frequently than laparoscopic myomectomies. This particular aspect could introduce a significant bias, as the variation in fertility rates in this scenario might be influenced by pre-operative conditions rather than the chosen surgical approach. In fact, certain studies have indicated that when dealing with myomas larger than 8 cm, the pregnancy rate undergoes a notable decrease from 50% to 30% [74]. Consequently, the question remains a subject of debate, with no definitive conclusions available. Another avenue of exploration, from a fertility standpoint, could involve the use of anti-adherence barriers.

### 4.3. Aesthetic Impact

Currently, the aesthetic impact plays a fundamental role both in the male and in the female population. Especially for a benign condition such as uterine fibroids, considerations regarding patients’ body image and their expectations must be taken into account. As reported in the literature, the impact of surgery could have consequences on the mental well-being of patients across different age groups and various types of pathologies [75]. From this point of view, endoscopy has traditionally been associated with superior aesthetic impact. While this holds true in many cases, some points of discussion have recently emerged, especially in cases of myomectomy conducted by suprapubic mini-laparotomy.

For instance, robotic surgery requires at least three to four incisions in the middle part of the abdomen, while the standard laparoscopy usually requires three to four incisions in the umbilicus and in the iliac fossa and suprapubic area. Even if the size of the incision is very small, especially in the case of mini laparoscopy [76], the scar is visible even many years after surgery, and the scar may be visible, for example, when wearing swimming suits.

A suprapubic mini-laparotomy incision consists of a very small incision (usually <5 cm) that is employed for myoma removal, minimizing the surgical impact and enhancing the aesthetic outcomes. This incision is small, but larger than the incision made for trocar positioning. Its unique feature lies in its low position in the suprapubic area, allowing it to be concealed beneath underwear [77].

However, this technique is not always feasible, especially in the case of huge myomas. The literature suggests a feasibility cut-off for this approach at myoma sizes exceeding 12 cm [78].

### 4.4. Comments on Data

In our review, 178,336 patients were studied, of which 163,886 underwent abdominal (open) myomectomy and 14,450 underwent laparoscopic or robotic myomectomy.

We report demographic, pre-operative, intra-operative, and post-operative parameters.

With regards to demographic factors, from our review, it emerged that age is not a parameter to decide the type of approach, as the average age of patients who underwent open myomectomy was 37.4 years, while the average age of patients who underwent minimally invasive myomectomy was 37.2 years. Also, the BMI remained comparable between the two groups, with patients undergoing open myomectomy averaging 24.6, while those undergoing minimally invasive myomectomy averaged 23.8. As for pre-operative parameters, we considered the number and size (defined as the maximum diameter in cm) of the fibroids, which were determined using ultrasound. Specifically, in the group of patients undergoing open myomectomy, the average number of fibroids was 5.4, with average dimensions of 8 cm. On the other hand, in the group of patients undergoing minimally invasive myomectomy, the average number of fibroids was found to be 3.7, with average dimensions of 7 cm. Both the numbers and the sizes of the fibroids were lower in the group of patients undergoing minimally invasive myomectomy compared to the fibroids of patients undergoing abdominal myomectomy. Strengthening this observation, our results indicated a statistically significant higher number of myomas in the laparotomic group across 10 studies [20,24,26,35,38,39,40,41,42,44].

This aspect is among the most crucial, as the sizes of myomas can influence extraction decisions. Furthermore, the number of myomas should be considered, along with their positions. In cases of multiple fibroids with submucosal components, the laparoscopic approach remains feasible but becomes more challenging, with an increased risk of blood loss. This factor may influence the decision to opt for a laparotomic approach. For example, in one of the most representative studies by Sandberg et al. [51], encompassing approximately 1000 patients, the differences in the numbers and weights of myomas were more than threefold (average number of 12.6 and weight of 592.75 g for the open approach vs. average number of 3.5 and weight of 263.4 g for the minimally invasive approach), and this result was significantly different. This highlights the limits of the laparoscopic approach in some cases, even if the surgeon’s experience remains one of the most important factors influencing surgical outcomes [79].

Regarding the characteristics of fibroids, in most cases, in both open and laparoscopic approaches, they are transmural myomas (501 operated on open vs. 559 operated minimally invasively). There is a slight prevalence of submucosal fibroids in the case of minimally invasive techniques (322 MI vs. 296 open). Similarly, most surgeons prefer minimally invasive techniques in the case of pedunculated fibroids (384 MI vs. 143 open). However, for fibroids located in other or multiple sites, abdominal surgery techniques are preferred. Some authors also considered the position of the fibroids. From the literature review, it emerged that open surgery was preferred for anteriorly located fibroids (39 vs. 26 operated with minimally invasive techniques), while laparoscopic and robotic techniques were preferred for fibroids located posteriorly and in the fundus (respectively 46 MI vs. 26 open and 6 MI vs. 1 open).

The estimated blood loss was documented in our review, revealing significant differences between the two groups in nine studies [20,24,26,35,36,39,42,43,44]. Specifically, the estimated blood loss in patients undergoing abdominal myomectomy averaged 378.33 mL, whereas in those undergoing the minimally invasive procedure, it averaged 217.4 mL. It is noteworthy that these differences could be related to the size and position of myomas, which in the laparotomic groups, as per the previously described characteristics, they might be more frequently associated with major bleeding. Nevertheless, the average bleeding did not reach the threshold of surgical complication (<500 mL) in either group, affirming the safety of both procedures.

The hospital stay duration was also assessed, with six studies showing a significantly lower duration for the minimally invasive approach [20,24,26,42,43,44]. This aligns with the existing literature, emphasizing one of the primary advantages of endoscopy, which is a reduction in the length of the hospital stay.

The pregnancy rate varied significantly for the different series. An average of 30.5% of patients experienced a pregnancy after a myomectomy. Among patients who underwent laparotomic surgery, the pregnancy rate after surgery was 29.7%, while for patients who underwent laparoscopic or robotic myomectomy, the pregnancy rate was 28.5%. Among these, the total percentage of deliveries was 22.6%. The rate of cesarean section for patients who underwent open myomectomy was 40%, while for patients who underwent laparoscopic or robotic myomectomy, it was 35.5%. Interestingly, the surgical approach did not appear to be significantly correlated with the pregnancy rate, as no statistically significant data were reported in any of the studies.

We also investigated the abortion rate, specifically noting it to be higher among patients who underwent abdominal surgery compared to those who underwent minimally invasive surgery (80.8% in the former case vs. 19.2%), excluding cases of biochemical pregnancy, ectopic pregnancy, and voluntary abortion [33]. The rate of preterm births was described in a few studies; in particular, according to some authors, it stood at around 2.3% in the case of patients undergoing minimally invasive surgery [47]. Few authors have reported the rate of complications during pregnancy in patients undergoing these surgical treatments. However, from our literature review, it emerged that this rate is higher in patients operated on via laparotomy (6%) compared to those operated on via laparoscopy (1.7%) [37].

Regarding post-operative parameters, the focus of our review was on immediate post-operative complications. On average, the percentage of complications in the group of patients undergoing abdominal myomectomy was 22.3%, while for patients operated on laparoscopically or robotically, it was 14.2%.

These are the most controversial data, as even though the average percentage of complications was lower for the endoscopic group, only one study reported a statistically significant difference (50% vs. 11.6% *p* < 0.001, respectively, for open surgery and endoscopy) [26]. However, it is worth noting that some studies did not report any statistical analyses. Another aspect that should be considered is the difference between major and minor complications. The incidence of minor complications such as fever or wound infection is more frequently associated with laparotomy. However, it is essential to recognize that despite their differences, both approaches exhibit low complication rates, demonstrating the safety of both procedures. Regarding the presence of intrauterine adhesions, this could be a parameter to better understand the complication incidence, but this finding was mentioned only in one of the articles considered in this review. Specifically, they appear to be present in 7.8% of open myomectomy cases and 8.6% of minimally invasive myomectomy cases [32].

## 5. Conclusions

The optimal surgical approach to myomectomy remains a highly debated topic. Currently, the literature lacks definitive answers to this question, although certain indications can be considered. The decision to approach the pathology endoscopically is fundamentally rooted in surgical experience and the availability of appropriate laparoscopic equipment. One of the main aspects that could be evaluated to decide the surgical approach involves the size and the number of fibroids. In cases of multiple and large fibroids with intramural and/or submucosal expansion, the open approach could be seriously taken into consideration. The mini-laparotomic approach remains a good alternative to endoscopy in terms of surgical outcomes and aesthetic results.

Regardless of the chosen approach, it is essential to highlight that fibroids should never be morcellated freely inside the abdomen. Instead, fibroid fragmentation should always be performed using containment systems.

The pregnancy rate seems not to be influenced by the type of surgical procedure. However, the different methodologies adopted, the different numbers of series, and the different enrollment criteria did not allow for incontrovertible indications. Therefore, further investigations are needed to shed more light on this matter.

## Figures and Tables

**Table 1:** Synthesis of selected studies.

Author, Year	Myomectomy	Mean Age	BMI	Number (N) and Dimension cm (D) of Fibroids	Fibroma’s Type and Location	Blood Loss (mL)	Hospital Stay (Days)	Clinical Pregnancy Rate after Surgery	Miscarriage Rate	Live Birth Rate	Complications Rate
Alharbi A. et al., 2020 [30]	Open (*n* = 213)	40.8 (*p*-value 0.135)	28.18 (*p*-value 0.289)	N 3.5 D 7.2 (*p*-value 0.628, *p*-value 0.005)	T 61 (*p*-value 0.000) S 47 (*p*-value 0.000) P 4 (*p*-value 0.000) MS 91 (*p*-value 0.000)	576.1 (*p*-value 0.003)	3.58 (*p*-value 0.000)	1.8% (*p*-value 0.786)	145 (*p*-value 0.892)	1.3% (*p*-value 0.673)	10.8%
	MI (*n* = 34)	42.0 (*p*-value 0.135)		N 4.2 D 5.3 (*p*-value 0.628, *p*-value 0.005)	T 1 (*p*-value 0.000) S 21 (*p*-value 0.000) P 2 (*p*-value 0.000) MS 10 (*p*-value 0.000)	333.2 (*p*-value 0.003)	1.64 (*p*-value 0.000)		27 (*p*-value 0.892)		2.9%
Bhave Chittawar P. et al., 2014 [31]	Open (*n* = 379)	31.75	NM	N 3 D 7	NM	NM	5.5	NM	NM	NM	0%
	MI (*n* = 429)				NM		4.7				36%
Bortoletto P. et al., 2022 [32]	Open (*n* = 52)	36.05 (*p*-value 0.046)	NM	N 3 D 5.5 (*p*-value 0.704 0.096)	S 103 (*p*-value 0.029)	NM	NM	1.25% (*p*-value 0.240)	NM	0	7.8% Intrauterine adhesions (*p*-value 0.800)
	MI (*n* = 70)										8.6% Intrauterine adhesions (*p*-value 0.800)
Boudova B. et al., 2019 [33]	Open (*n* = 7)	34.4	NM	N 2.5 D 5.7	NM	NM	NM	63.6%	66.7% (8 miscarriages, 1 ectopic pregnancy, 4 terminations of pregnancies on patient’s request)	33.3% (CS 8 VB 2)	25.7% Re-interventions for radical treatment
	MI (*n* = 30)				NM						
Catanese A. et al., 2022 [34]	Open (*n* = 99)	38.2 (*p*-value 0.42)	21.7 (*p*-value 0.86)	N 1 D 10 (*p*-value < 0.0001, <0.0001)	NM	300 (*p*-value < 0.0001)	3 (*p*-value < 0.0001)	NM	NM	NM	0% (*p*-value > 0.99)
	MI (*n* = 361)	37.6 (*p*-value 0.42)	22 (*p*-value 0.86)	N 1 D 7 (*p*-value < 0.0001, <0.0001)		200 (*p*-value < 0.0001)	3 (*p*-value < 0.0001)				Early (>30 days after surgery) 0.83% Late (>30 days after surgery) 0% (*p*-value > 0.99)
Chin H. et al., 2014 [35]	Open (*n* = 1)	42	NM	N 3 D 6.5	NM	NM	NM	NM	NM	NM	0%
	MI (*n* = 2)	34		N 3 D 4.8							50%
D’Silva E.C. et al., 2018 [36]	Open (*n* = 22)	33.3 (*p*-value 0.705)	NM	N 5 D 10 (*p*-value < 0.001)	NM	1290.5 (*p*-value < 0.001)	3 (*p*-value < 0.001)	NM	NM	NM	50% (*p*-value 0.001)
	MI (*n* = 67)	34.0 (*p*-value 0.705)		N 3 D 7.5 (*p*-value < 0.001)		406.6 (*p*-value < 0.001)	2 (*p*-value < 0.001)				11.6% (*p*-value 0.001)
Flyckt R. et al., 2016 [37]	Open (*n* = 81)	34.1 (*p*-value 0.77)	28.2 (*p*-value 0.20)	NM	NM	NM	NM	66.7% (*p*-value 0.39)	12% (total: 134) (*p*-value 0.12)	5.2% (*p*-value 0.30)	NM
	MI (*n* = 53)	33.7 (*p*-value 0.77)	27.1 (*p*-value 0.20)					50% (*p*-value 0.39)	4% (total: 118) (*p*-value 0.12)	4.3% (*p*-value 0.30)	
Frost A. et al., 2021 [38]	Open (*n* = 106,520)	35.5 (*p*-value 0.001)	NM	NM	NM	NM	2.66	NM	NM	NM	NM
	MI (*n* = 8330)	45.5 (*p*-value 0.001)					2.48				
Gil Y. et al., 2020 [39]	Open (*n* = 52,917)	33.3 (*p*-value < 0.001)	7.7% BMI > 25 (*p*-value 0.08)	NM	NM	NM	NM	NM	NM	NM	Uterine rupture 0.4%
	MI (*n* = 1229)		6.8% BMI > 25 (*p*-value 0.08)								
Grainger T. et al., 2023 [40]	Open (*n* = 21)	42 (*p*-value 0.25)	NM	NM	NM	NM	NM	100% (*p*-value 0.16)	NM	100% CS 52% VB 48%	Uterine rupture 0%
	MI (*n* = 25)									100% CS 48% VB 52%	Uterine rupture 0%
Kim, H. et al., 2018 [41]	Open (*n* = 13)	38.1 (*p*-value 0.437)	23.4 (*p*-value 0.103)	N 13.5 D 8.1 (*p*-value 0.920, 0.125)	NM	323.1 (*p*-value 0.724)	3.5 (*p*-value 0.003)	NM	NM	NM	30.8% (*p*-value >0.999)
	MI (*n* = 13)	37 (*p*-value 0.437)	21.6 (*p*-value 0.103)	N 13.7 D 6.8 (*p*-value 0.920, 0.125)		219.2 (*p*-value 0.724)	2.5 (*p*-value 0.003)				30.8% (*p*-value > 0.999)
Iavazzo C. et al., 2014 [42]	Open (*n* = 135)	NM	NM	NM	NM	257.3	2.96	NM	NM	NM	NM
	MI (*n* = 102)					283.9	3.62				
Iavazzo C. et al., 2016 [43]	Open (*n* = 1287)	37.6	25.8	N 3.3 D 6.7	NM	252.1	2.59	5.9%	NM	NM	17.1%
	MI (*n* = 895)	35.6	24.9	N 2.5 D 6.4		182.1	1.53	13.3%			6%
Jayakumaran J. et al., 2017 [44]	Open (*n* = 797)	NM	NM	NM	NM	309.6	3.32	NM	NM	NM	NM
	MI (*n* = 1108)					200.9	1.75	9.6%			
Kotani Y. et al., 2018 [45]	Open (*n* = 279)	36 (*p*-value < 0.001)	22.1 (*p*-value 0.061)	N 6.5 D 9 (*p*-value < 0.001, <0.001)	NM	554	11.7	15.1%	NM	NM	NM
	MI (*n* = 474)	37.6 (*p*-value < 0.001)	21.7 (*p*-value 0.061)	N 3.7 D 7 (*p*-value < 0.001, <0.001)		207 (*p*-value < 0.001)	3.5 (*p*-value < 0.001)	14.6% (*p*-value < 0.853)			
Lee S.R. et al., 2020 [46]	Open (*n* = 151)	38.1 (*p*-value 0.966)	23.5 (*p*-value 0.408)	N 4 D 11.2 (*p*-value 3.51 × 10^−5^, 0.233)	S 10 (*p*-value 7.96 × 10^−4^) O 141 (*p*-value 7.96 × 10^−4^)	297.1 (*p*-value 0.009)	4.13 (*p*-value 8.74 × 10^−13^)	NM	NM	NM	54.3% (*p*-value 5.16 × 10^−6^)
	MI (*n* = 126)	38.1 (*p*-value 0.966)	23 (*p*-value 0.408)	N 3 D 10.8 (*p*-value 3.51 × 10^−5^, 0.233)	S 28 (*p*-value 7.96 × 10^−4^) O 89 (*p*-value 7.96 × 10^−4^)	368.4 (*p*-value 0.009)	2.68 (*p*-value 8.74 × 10^−13^)				26.1% (*p*-value 5.16 × 10^−6^)
Metwally M. et al., 2020 [47]	Open (*n* = 91)	NM	NM	NM	T 26 S 21 (MANCA *p*-VALUE!)	NM	NM	45%	16%	36% (CS 28%)	NM
	MI (*n* = 86)							44.5%	8.1%	32.5% (CS 23%)	
Ming X. et al., 2019 [48]	Open (*n* = 313)	37.2 (*p*-value 0.418)	22.1 (*p*-value 0.821)	N 1.5 D 6.7 (*p*-value 0.626, < 0.001)	T 194 (*p*-value 0.302) S 20 (*p*-value 0.302) O 99 (*p*-value 0.302)	NM	NM	21.8% (*p*-value 0.121)	NM	NM	NM
	MI (*n* = 83)	37.7 (*p*-value 0.418)	22.2 (*p*-value 0.821)	N 1.4 D 5.2 (*p*-value 0.626, < 0.001)	T 46 (*p*-value 0.302) S 10 (*p*-value 0.302) O 27 (*p*-value 0.302)			30.3% (*p*-value 0.121)	NM		
Morales H.S.G. et al., 2022 [49]	Open (*n =* 21)	36.9 (*p*-value 0.287)	25.6 (*p*-value 0.049)	N 9.2 D 9.7 (*p*-value 0.000, 0.004)	T 33.3% (*p*-value 0.069)	502.9 (*p*-value 0.097)	2.1 (*p*-value 0.525)	23.8%	1 (*p*-value 0.744)	40% (*p*-value 0.744)	4%
	MI (*n =* 48)	34.7 (*p*-value 0.287)	24 (*p*-value 0.049)	N 3.2 D 4.9 (*p*-value 0.000, 0.004)	T 69.2% (*p*-value 0.069)	215.3 (*p*-value 0.097)	1.94 (*p*-value 0.525)	37.45%	2 (*p*-value 0.744)	38.8% (*p*-value 0.744)	0%
Ozbaşlı E. et al., 2021 [50]	Open (*n* = 73)	38.8 (*p*-value 0.590)	24.7 (*p*-value 0.003)	N 4 D 5.5 (*p*-value 0.002, <0.001)	P 1 (*p*-value 0.356) A 5 (*p*-value 0.356) Po 2 (*p*-value 0.356) MS 64 (*p*-value 0.356) F 1 (P-value 0.356)	100 (*p*-value 0.098)	1 (56.2%) (*p*-value 0.013)	NM	NM	NM	1.4% (*p*-value 0.800)
	MI (*n* = 154)	38.3 (*p*-value 0.590)	22.9 (*p*-value 0.003)	N 3 D 7.5 (*p*-value 0.002, <0.001)	P 6 (*p*-value 0.356) A 4 (*p*-value 0.356) Po 7 (*p*-value 0.356) MS 133 (*p*-value 0.356) F 6 (*p*-value 0.356)	135 (*p*-value 0.098)	1 LM (64.8%) 2 RM (54.5%) (*p*-value 0.013)		NM		2.6% (*p*-value 0.800)
Sandberg E.M. et al., 2015 [51]	Open (*n* = 235)	39.8 (*p*-value 0.060)	27.5 (*p*-value 0.970)	N 12.6 D 592.75 g (weight) (*p*-value < 0.001, <0.001)	T 98 (*p*-value 0.002) P 125 (*p*-value 0.245) S 66 (*p*-value 0.225)	267.2	2.15	NM	NM	NM	NM
	MI (*n* = 731)	40.3 (*p*-value	26.7 (*p*-value 0.970)	N 3.5 D 263.4 g (weight) (*p*-value < 0.001, <0.001)	T 394 (*p*-value 0.002) P 373 (*p*-value 0.245) S 131 (*p*-value 0.225)	181.5	0.58				
Strong S. M. et al., 2020 [52]	Open (*n* = 58)	40 (*p*-value < 0.001)	27 (*p*-value 0.288)	N 6 D 10 (*p*-value < 0.01, <0.001)	T 59 S 29 P 13 MS 13 (*p*-VALUE MANCANTE!)	400 (*p*-value < 0.01)	2 (*p*-value < 0.01)	NM	NM	NM	3.2%
	MI (*n* = 93)	37 (*p*-value < 0.001)	27 (*p*-value 0.288)	N 3 D 14 (*p*-value < 0.01, <0.001)		200 (*p*-value < 0.01)	1 (*p*-value < 0.01)				5.2%
Tian Y. et al., 2021 [53]	Open (*n* = 63)	33.0 (*p*-value 0.491)	23.6 (*p*-value 0.401)	N 3.2 D 4 (*p*-value 0.792)	T 27 (*p*-value 0.717) O 36 (*p*-value 0.717) MS 36 (*p*-value 0.717)	140.1 (*p*-value < 0.001)	10.3 (*p*-value < 0.001)	NM	NM	NM	23.8%
	MI (*n* = 63)	33.4 (*p*-value 0.491)	23.3 (*p*-value 0.401)	N 3.2 D 3.9 (*p*-value 0.792)	T 29 (*p*-value 0.717) O 34 (*p*-value 0.717) MS 38 (*p*-value 0.717)	63.7 (*p*-value < 0.001)	6.9 (*p*-value < 0.001)				9.5%
Tinelli A. et al., 2013 [54]	Open (*n =* 58)	36.4 (*p*-value 0.4991)	24.5 (*p*-value 0.035)	N multiple 51.8% D 6.5 (*p*-value 0.3864, 0.0005)	T 36 (*p*-value 0.0643) O 22 (*p*-value 0.0643) A 34 (*p*-value < 0.05) Po 24 (*p*-value < 0.05)	105 (*p*-value 0.00001)	3.5 (*p*-value 0.00001)	NM	NM	NM	60.3%
	MI (*n =* 66)	35.7 (*p*-value 0.4991)	23.4 (*p*-value 0.035)	N single 56% D 7.6 (*p*-value 0.3864, 0.0005)	T 30 (*p*-value 0.0643) O 36 (*p*-value 0.0643) A 22 (*p*-value < 0.05) Po 39 (*p*-value < 0.05)	65 (*p*-value 0.00001	1.5 (*p*-value 0.00001)	NM		NM	16.6%

MI: minimally invasive, N: numbers, D: dimension, NM: not mentioned, CS: Cesarean section, VB: vaginal birth; T: transmural, S: submucosal, P: pedunculated, MS: multiple sites, O: others, A: anterior, Po: posterior, F: fundus.

## Data Availability

All data are available by reference list of the article selected for this review.
